# Depolarizing Channel Mismatch and Estimation Protocols for Quantum Turbo Codes

**DOI:** 10.3390/e21121133

**Published:** 2019-11-20

**Authors:** Josu Etxezarreta Martinez, Pedro M. Crespo, Javier Garcia-Frías

**Affiliations:** 1 Department of Basic Sciences, Tecnun—University of Navarra, 20018 San Sebastian, Spain; pcrespo@tecnun.es; 2 Department of Electrical and Computer Engineering, University of Delaware, Newark, DE 19716, USA; jgf@udel.edu

**Keywords:** quantum error correction, turbo codes, depolarizing channel, estimation

## Abstract

Quantum turbo codes (QTC) have shown excellent error correction capabilities in the setting of quantum communication, achieving a performance less than 1 dB away from their corresponding hashing bounds. Decoding for QTCs typically assumes that perfect knowledge about the channel is available at the decoder. However, in realistic systems, such information must be estimated, and thus, there exists a mismatch between the true channel information and the estimated one. In this article, we first heuristically study the sensitivity of QTCs to such mismatch. Then, existing estimation protocols for the depolarizing channel are presented and applied in an off-line manner to provide bounds on how the use of off-line estimation techniques affects the error correction capabilities of QTCs. Finally, we present an on-line estimation method for the depolarizing probability, which, different from off-line estimation techniques, neither requires extra qubits, nor increases the latency. The application of the proposed method results in a performance similar to that obtained with QTCs using perfect channel information, while requiring less stringent conditions on the variability of the channel than off-line estimation techniques.

## 1. Introduction

Quantum turbo codes have been demonstrated to be a promising family of quantum error correction codes (QECC) with performances as close as 0.3 dB to their hashing bounds. Proposed as the serial concatenation of quantum convolutional codes (QCC) in [1], their original performance lacked the closeness to the hashing limit that was desired when the classical-to-quantum isomorphism [2] was applied in order to adapt the classical turbo codes originally constructed by Berrou et al. in [3] to the quantum realm. Entanglement-assistance (EA) was invoked in [4] as the resource needed in order to overcome the fact that unassisted QCCs cannot be both non-catastrophic and recursive simultaneously [5]. It turns out that inner encoders that have those two properties are the key for QTCs in order to show not only a minimum distance that grows linearly with the blocklength, but also a decoding algorithm that achieves convergence. The application of this technique narrowed the gap that the QTCs present with respect to their hashing bounds and made them one of the most promising families of QECCs. Since then, the field of turbo codes in the quantum paradigm has been extensively studied, starting from the usage of extrinsic information transfer (EXIT) chart techniques by Babar et al. in [6] with the aim of optimizing the QTCs to have a performance that lies even nearer to the hashing bound. Afterwards, quantum irregular convolutional codes (QIRCC) and their entanglement-assisted counterparts were proposed in [7,8] as outer codes for lowering the error floors that the optimized codes of [6] show. Quantum unity rate codes (QURC) were proposed as inner encoders in [9] for constructing QTCs with higher rates. Recently, the construction of interleavers with the aim of lowering the error floors of QTCs was addressed in [10] by Etxezarreta et al., where *S*-random, JPL, and Welch–Costas interleavers were considered to improve the error correction capability of the codes.

The QTC decoder, consisting of two serially concatenated soft-input soft-output (SISO) decoders, uses channel information as the input in order to engage in degenerate decoding and to estimate the most probable error coset to correct the corrupted quantum state. Previous works on QTCs [1,2,3,4,5,6,7,8,9,10,11] work under the assumption that such a decoder has perfect channel knowledge, that is the system is able to estimate the depolarizing probability of the quantum channel perfectly. However, this scenario is not realistic, since in reality, the decoder should work with estimates of the depolarizing probability rather than with the exact value. The effect of channel mismatch on QECCs has been studied for quantum low density parity check (QLDPC) codes in [12,13], which showed that such codes are pretty insensitive to the errors introduced by the imperfect estimation of the depolarizing probability and proposed methods to improve the performance of such QECCs when the depolarizing probability is estimated. For classical parallel turbo codes, several studies showed their insensitivity to SNR mismatch [14,15,16,17]. In such works, it was found that the performance loss of parallel turbo codes is small if the estimated SNR is above the actual value of the channel. However, QTCs can only be constructed as serially concatenated convolutional codes, and classical studies about channel information mismatch for such codes [18,19] showed that such structures are more susceptible to channel identification mismatch, suffering a significant performance loss if the SNR is either overestimated or underestimated. The authors in [18] justified the increase in sensitivityby pointing out that in parallel turbo codes, the channel estimates are fed to both decoders, which distributes the errors between the two constituent codes. However, for serial turbo codes, the errors are not as evenly spread as the channel information is just used in the inner decoding stage. This strongly suggests that (serially concatenated) QTCs will be more sensitive to depolarizing probability mismatch.

In this paper, we first analyze the performance loss incurred by QTCs due to the channel’s depolarizing probability mismatch. Then, we study how different off-line estimation protocols affect the overall QTCs’ performance and provide some heuristic guidelines to selecting the number of quantum probes required to have an estimate of the depolarizing channel that keeps the performance degradation of such codes low. Finally, we propose an on-line estimation procedure by utilizing a modified version of the turbo decoding algorithm that allows, at each decoding iteration, estimating the channel information to be fed to the inner SISO decoder. The procedures used throughout this paper to analyze the performance of the methods presented are of a heuristical nature.

The remainder of this paper is organized as follows: Section 2 presents the depolarizing channel and the QTC system model; Section 3 presents the sensitivity of the QTC decoder with respect to the depolarizing probability mismatch; Section 4 presents existing off-line estimation methods for the depolarizing channel and obtains bounds for the performance of QTCs when such estimators are applied; Section 4 also describes the proposed on-line estimation method and investigates its resulting performance; finally, Section 5 provides the conclusions reached in this paper.

## 2. Preliminaries: The Quantum Depolarizing Channel and Quantum Turbo Codes

### 2.1. Quantum Depolarizing Channel

In this study, we consider that the quantum channel acting on an arbitrary quantum state ρ is the depolarizing channel (The depolarizing channel) is a widely applied channel model used to represent the decoherence effects that produce errors in quantum information [1,2,3,4,6]. $N_{D}$ defined as:

(1) $$N_{D}\left(\rho ,\epsilon \right)=\left(1-\epsilon \right)\rho +\epsilon \frac{I}{2}=\left(1-\frac{3}{4}\epsilon \right)\rho +\frac{\epsilon }{4}\left(X\rho X+Y\rho Y+Z\rho Z\right),$$

where ϵ is the probability that the quantum state density matrix is transformed to the maximally mixed state $\frac{I}{2}$ by the operation of the channel and X,Y,Z are the Pauli matrices. For decoding purposes, the depolarizing channel is often parameterized as:

(2) $$N_{D}\left(\rho ,p\right)=\left(1-p\right)\rho +\frac{p}{3}\left(X\rho X+Y\rho Y+Z\rho Z\right),$$

where *p* is called the depolarizing probability. Such a channel acts independently on each of the qubits transmitted through the channel, inflicting a bit-flip, phase-flip, or both bit- and phase-flip error on each of the qubits with probability p/3. Note that both Expressions (1) and (2) are equivalent by setting $p=\frac{3}{4}\epsilon$. Unless otherwise stated, when we mention the depolarizing channel in the sequel, we will refer to the transformation given by (2).

### 2.2. Quantum Turbo Codes

**Figure 1:**
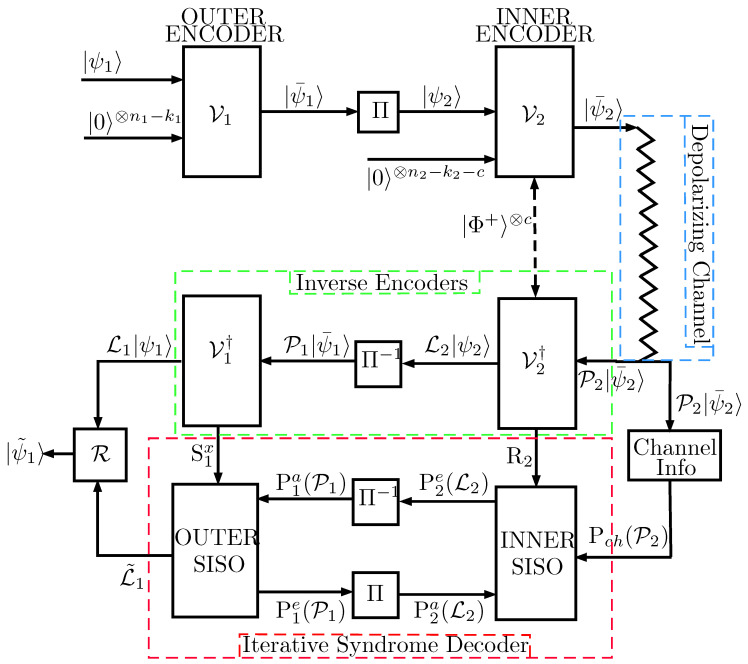
Schematic of the QTC. Note that the *c* pre-shared EPR pairs ${|\Phi \rangle }^{+}$ are needed for the inner encoder to be both recursive and non-catastrophic [4]. $P_{i}^{a}\left(.\right)$ and $P_{i}^{e}\left(.\right)$ denote the a priori and extrinsic probabilities related to each of the SISO decoders used for turbo decoding.

The quantum turbo codes considered in this paper consist of the interleaved serial concatenation of unassisted QCCs acting as outer codes and entanglement-assisted QCCs inner codes. Figure 1 presents the full schematic representation of such a quantum error correction system. The *k* input logical qubits that compose the information word $|\psi_{1}\rangle$ are first fed to the outer $\left[n_{1},k_{1},m_{1}\right]$ unassisted convolutional encoder $V_{1}$ and encoded into $n^{\prime }=k\frac{n_{1}}{k_{1}}$ physical qubits with the help of $\left(n_{1}-k_{1}\right)$ ancilla qubits and *m* memory qubits. The $n^{\prime }$ physical qubits that form the codeword $|{\bar{\psi }}_{1}\rangle$ generated by the first encoder are then passed through a quantum interleaver П before being input to the inner convolutional encoder $V_{2}$. Such an encoder is an $\left[n_{2},k_{2},m_{2},c\right]$ entanglement-assisted encoder that encodes the interleaved sequence of $n^{\prime }$ qubits $|\psi_{2}\rangle$ into the codeword $|{\bar{\psi }}_{2}\rangle$ of length $N=n^{\prime }\frac{n_{2}}{k_{2}}=k\frac{n_{2}}{k_{2}}\frac{n_{1}}{k_{1}}$, aided by $\left(n_{2}-k_{2}-c\right)$ ancilla qubits, *c* pre-shared EPR pairs, and $m_{2}$ memory qubits. Codeword $|{\bar{\psi }}_{2}\rangle$ is then transmitted through a quantum depolarizing channel with depolarizing probability *p* inflicting an *N*-qubit Pauli error $P_{2}\in G_{N}$ on the codeword. The depolarizing channel is independently applied to each of the qubits of the stream $|{\bar{\psi }}_{2}\rangle$, and consequently, each of the qubits experiences a bit-flip (X operator) with probability p/3, a phase-flip (Z operator) with probability p/3, or a combination of both (Y operator) with probability p/3, as is described in Equation (2).

At the output of the depolarizing channel, the state $P_{2}|{\bar{\psi }}_{2}\rangle$ is fed to the inverse of the inner encoder $V_{2}^{†}$, which outputs the decoded state $L_{2}|\psi_{2}\rangle$, where $L_{2}\in G_{n^{\prime }}$ refers to the logical error suffered by the decoded state due to the operation of the channel; and the classical syndrome bits $R_{2}=\left(S_{2}^{x},E_{2}^{x,z}\right)$ obtained from *Z* basis measurements on the ancilla qubits and Bell measurements on the pre-shared EPR pairs. The corrupted logical qubits are then passed through a de-interleaver ${П}^{-1}$ resulting in the state $P_{1}|{\bar{\psi }}_{1}\rangle$, which is supplied to the inverse of the outer encoder $V_{1}^{†}$. The resulting output is the state $L_{1}|\psi_{1}\rangle$, which corresponds to the information quantum state corrupted by a logical error $L_{1}\in G_{k}$; and the classical syndrome bits $S_{1}^{x}$ obtained after measuring the ancilla qubits on the *Z* basis. The classical syndromes $R_{2}$ and $S_{1}^{x}$, obtained in the inverse decoders $V_{2}^{†}$ and $V_{1}^{†}$, respectively, are then provided to the iterative syndrome decoder consisting of two serially concatenated SISO decoders, as shown in Figure 1. Based on $R_{2}$ and $S_{1}^{x}$, as well as the channel polarization probability $P_{ch}\left(P_{2}\right)$, both SISO decoders engage in degenerate iterative decoding [1,4] to estimate the most likely error coset ${\tilde{L}}_{1}$ that has corrupted the information quantum state. Based on such an estimation, a recovery operation R is applied to the corrupted state $L_{1}|\psi_{1}\rangle$, yielding the recovered output $|{\tilde{\psi }}_{1}\rangle$.

As already mentioned, the aim of this paper is to study the QTCs’ performance loss due to imperfect channel information and to assess how different estimation protocols perform on such codes. To that end, the inner and outer QCCs used for the quantum turbo code in Figure 1 are the EXIT-optimized codes designed in [6] (see their parameters in Table 1). Such a configuration was extensively studied in [6] under an exact channel information assumption. For that reason, we will utilize them as benchmarks in this paper.

From the parameters in Table 1, the QTCs used in this paper have a rate 1/9 with an entanglement consumption rate of 6/9. The noise limit $p^{*}$ can be found from the entanglement-assisted hashing bound, which for such parameters is $p^{*}=0.3779$ [4].

## 3. Quantum Turbo Decoder Performance with Depolarizing Probability Mismatch

In this section, we study the performance sensitivity of quantum turbo codes when transmitting over the depolarizing channel, assuming that their turbo decoders have a mismatched value $\hat{p}$ of the actual channel depolarizing probability *p*. To that end, Monte Carlo computer simulations of the coding system shown in Figure 1 were carried out. The blocklength of the QTC was set to k=1000 input logical qubits, and the figure of merit used to test the performance was the probability that at least one qubit of the received block was incorrectly decoded, that is the word error rate (WER).

Similar to [4,10] and in order to perform the numerical simulations, an *n*-qubit error was randomly generated for each block in each transmission round as defined in (2). At the decoder, the syndromes $R_{2}$ and $S_{1}^{x}$ were first computed, and the iterative turbo decoding algorithm ran until either the hard decisions of the estimated logical errors were the same as in the previous iteration or the number of iterations reached 15. The turbo decoding algorithm was fed with a mismatched value of the depolarizing probability $\hat{p}$ for the channel information $P_{ch}\left(P_{2}\right)$.

As explained in [20] and under the assumption that error events are independent, the number of transmitted blocks, $N_{blocks}$, required to estimate WER within a 95% confidence interval of about $\left(0.8\hat{\mathrm{WER}},1.25\hat{\mathrm{WER}}\right)$, where $\hat{\mathrm{WER}}$ refers to the empirically estimated value for the WER, is:

(3) $$N_{blocks}=\frac{100}{\mathrm{WER}}.$$

**Figure 2:**
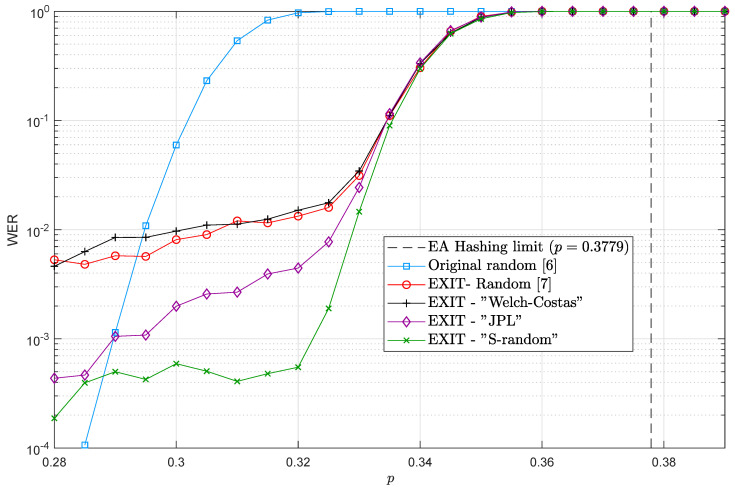
Word error rate (WER) performance curves for the 1/9-QTCs in Table 1 when different interleavers are used. Perfect channel knowledge is assumed.

For completeness in the exposition, we first present the WER performance of the simulated QTC assuming perfect channel state information at the QTC decoder. This is shown in Figure 2, where the WER versus the actual channel depolarizing probability *p* is plotted for several classes of interleavers (refer to [10]). In the sensitivity study that follows, we consider the EXIT-random interleaver for our QTC and later present the impact of the choice of interleavers for this particular problem. We will focus on the relevant channel depolarizing probability interval, p∈[0.3,0.35].

**Figure 3:**
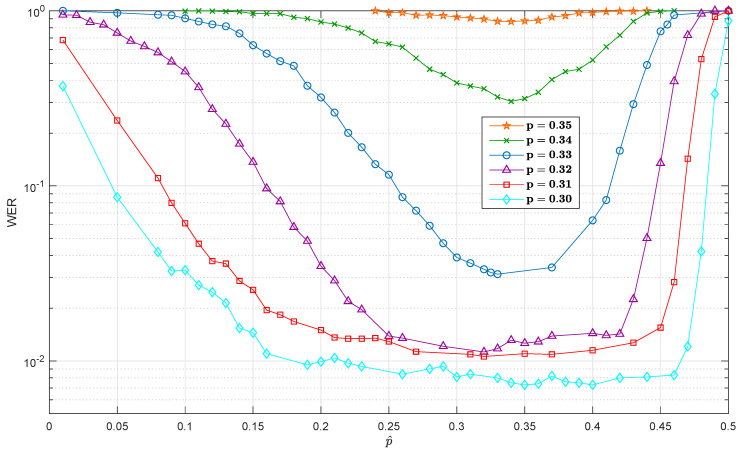
WER variation for QTCs using an EXIT-random interleaver as a function of the mismatched depolarizing probability $\hat{p}$. Each one of the curves corresponds to a value of the actual channel depolarizing probability. The QTCs considered here have an entanglement consumption rate of 6/9.

Figure 3 shows the WER versus the mismatched depolarizing probability $\hat{p}$ fed to the turbo decoder. Six curves are plotted in the same figure, corresponding to six different depolarizing channels with probabilities p∈{0.35,0.34,0.33,0.32,0.31,0.30}. Figure 3 shows the strong relationship between the mismatch sensitivity and the actual channel depolarizing probability, *p*. The smaller *p* is, the wider the flat (We define the flat region as the zone in the sensitivity curve where the WER performance is not considerably degraded as a consequence of the mismatch.) region is. As expected, the ability of a QTC to correct errors improved as the depolarizing probability of the channel *p* decreased. Comparing these results to the mismatched sensitivity work presented in [12,13] for QLDPCs, we can conclude that QLDPCs were more robust against channel mismatch than quantum turbo codes, since QLDPCs had a behavior flatter than that of QTCs when the depolarizing probability was under-estimated. This type of behavior can be explained by comparing QTCs with classical turbo codes. In the realm of classical turbo codes, the same kind of (Note that in the classical realm, mismatch means that the noise variance of the channel has to be estimated.) behavior is encountered for parallel and serial turbo codes [14,15,16,17]. That is, classical parallel turbo codes present a sensitive region flatter than serial ones [18,19] when the variance of the AWGN channel is under-estimated, which may be explained by the fact that in serial configurations, contrary to what happens in parallel turbo codes, the channel state information is only used in the inner decoding stage, and thus, errors due to mismatch only appear at the first decoding stage and propagate to the outer decoder in the turbo decoder.

### Interleaver Impact

The authors in [10] showed that the selection of the interleaver plays a central role in the design of QTCs due to their ability to reduce the error floors in such codes. This raises the question of whether different classes of interleavers may also influence the sensitivity behavior of the QTC under channel mismatch. To investigate this possibility, the previous simulation was also carried out for the following two interleavers, which according to Figure 2 were the ones with the lowest error floors:

- *S*-random interleaver with parameter S=25 and
- JPL interleaver.

**Figure 4:**
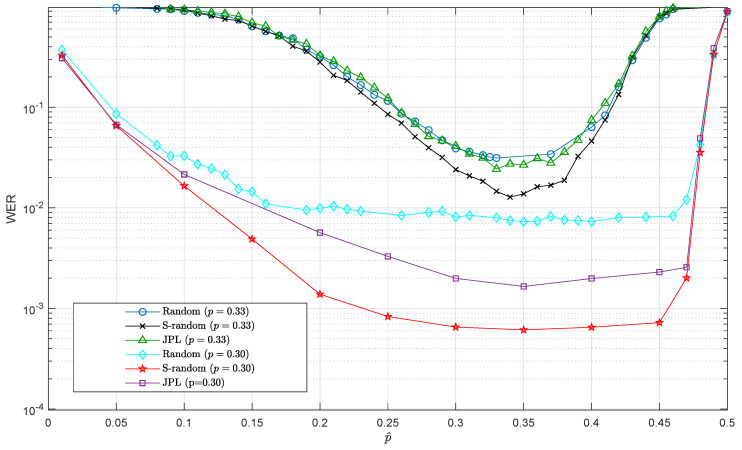
WER variation for the QTCs with three different interleavers as a function of the mismatched depolarizing probability $\hat{p}$. The true depolarizing probabilities selected are p=0.33 and p=0.30.

The next set of simulations was performed by setting the depolarizing channel probability to p=0.33 and p=0.30, which by looking at the curves in Figure 2 were the values of *p* where the WERs started to diverge in the waterfall region and where the WERs lied in the error floor region, respectively. The results are plotted in Figure 4. Note that in the same figure, we also included the curves corresponding to the EXIT-random interleaver previously plotted in Figure 3. Observe that the curves of WER versus $\hat{p}$ were very similar for the all three interleavers under consideration as the flat region for each of them had approximately the same width. It is true that for p=0.30, both the JPL and the *S*-random interleavers presented a lower WER in the flat region, but that was due to the fact that their error floor was lower. Therefore, we concluded that even though the selection of the interleaver had a significant impact on the resulting error floor of the QTC, the influence that interleavers had on the mismatch sensitivity was negligible.

The explanation for this invariance of the mismatch sensitivity to the interleaver choice is that the depolarizing probability was used only in the inner SISO decoder. This means that the extra errors that appeared in the decoding due to the mismatched depolarizing probability will be generated in the inner part of the turbo decoder, and they will propagate through the interleaver to the outer convolutional decoder. Consequently, even if using the *S*-random or the JPL interleaver means a higher error correction capability in the error floor region, the sensitivity of the overall decoder to channel mismatch, defined by the inner decoder, will not be affected by interleaver choice.

In the next section, we discuss the performance of QTCs over depolarizing channels when the depolarizing probability (2) is unknown and has to be estimated.

## 4. Estimating the Depolarizing Probability

The problem of quantum channel identification (quantum process tomography [21]) is of fundamental relevance for several quantum information processing tasks. In the context of quantum error correction, it is often assumed that the decoder knows exactly the quantum channel. Unfortunately, such an assumption may not be valid as the channel is usually unknown. Consequently, the QTC decoder should be provided with an estimate of the channel. In our case, since the quantum channel acting on an arbitrary quantum state ρ was the depolarizing channel $N_{D}$, the decoder QTC should be fed with an estimate of the channel depolarizing probability, *p*. It will be assumed that *p* remains constant for at least the duration of a QTC channel block (i.e., during 9000 qubits for the codes in this paper). Two types of estimation frameworks were considered: off-line and on-line.

### 4.1. Off-Line Estimation Framework

In this framework, the channel depolarization probability is estimated before block transmission can begin. Note that if the channel remains constant during the transmission of all blocks (i.e., the quantum channel is unknown but constant) then the QTC rate loss will be asymptotically negligible as the number of transmitted blocks increases. On other hand, if the channel varies at every transmitted block (but remains constant during a block), this off-line estimation method substantially reduces the overall rate of the QTC.

#### 4.1.1. Quantum Channel Estimation

**Figure 5:**
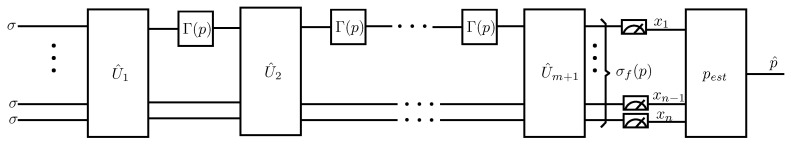
General channel identification method for a *p* parameter dependent quantum channel Γ(p) [22]. For the scenario of this paper, $\Gamma \left(p\right)=N_{D}$. This general scenario uses *n* quantum probes σ and *m* channel invocations. $U_{1},\cdots ,U_{m+1}$ are parameter independent transformations that can be effectively selected to estimate *p*. $p_{est}$ is an estimator function that uses the classical information $\left(x_{1},\cdots ,x_{n}\right)$ obtained from measuring the output quantum state $\sigma_{f}\left(p\right)$ in order to get the estimate $\hat{p}$.

In general, quantum channel identification requires suitably prepared quantum states to be subjected to the operation of the quantum channel Γ(p), [22]. Figure 5 shows the general estimation scheme used for channel identification. It used *n* equal quantum probes σ that were then passed through unitary operators $U_{i}$ while being submitted to the action of the target quantum channel *m* times. The output quantum state $\sigma_{f}\left(p\right)$, which depends on the parameter *p*, was then measured qubit-wise to obtain the classical information $\left(x_{1},\cdots ,x_{n}\right)$ that will be used to get the estimate $\hat{p}=p_{est}\left(x_{1},\cdots ,x_{n}\right)$.

Since the estimation of *p* depends on the measurements obtained from the state $\sigma_{f}\left(p\right)$ and such measurements in quantum physics are statistically distributed, the depolarization probability $\hat{p}$ will be a random variable. Consequently, the aim of channel identification is to chose a procedure that yields estimations of *p* with minimal fluctuations around the actual parameter value. The way to quantify these fluctuations is by computing the variance of the estimation, $\mathrm{var}\left(\hat{p}\right)=E\left\{{\left(\hat{p}-p\right)}^{2}\right\}$, where it is assumed that the estimator is unbiased, i.e., $E\{\hat{p}\}=p$. Obviously, the variance will depend on the actual choice of the estimator used. For the purposes of this section, we will assume that the estimation scheme achieves the information-theoretical optimal performance, that is the quantum Cramér–Rao bound. In that sense, the results in this section will be bounds for off-line channel estimation.

The quantum Cramér–Rao bound states that the variance on any estimator is bounded below as [22]:

(4) $$\mathrm{var}\left(\hat{p}\right)\geq \frac{1}{J(p)},$$

where J(p) is the quantum Fisher information, which depends only on the output quantum state $\sigma_{f}\left(p\right)$, but neither on the choice of measurement (Note that the classical Fisher information F(p) depends on the choice of measurement, but as F(p)≤J(p), the Cramér–Rao bound can be rewritten as a function of the measurement-independent quantum Fisher information [22].) $\left(x_{1},\cdots ,x_{n}\right)$, nor on the applied estimation scheme (refer to Figure 5). It is calculated as:

(5) $$J\left(p\right)=\mathrm{Tr}[\sigma_{f}\left(p\right){\hat{L}}^{2}\left(p\right)],$$

where $\hat{L}\left(p\right)$ is the symmetric logarithmic derivative (SLD) defined implicitly via:

(6) $$\frac{\partial \sigma_{f}\left(p\right)}{\partial p}=\frac{1}{2}\left[\hat{L}\left(p\right)\sigma_{f}\left(p\right)+\sigma_{f}\left(p\right)\hat{L}\left(p\right)\right].$$

Quite generally, there exists a measurement procedure that asymptotically attains the quantum Cramer–Rao bound [23]. From the fact that $\sigma_{f}\left(p\right)$ depends only on the resources used to test the channel, the same dependence will hold for J(p), and in turn for the quantum Cramér–Rao bound. Note that there are many possible combinations of these resources, which will lead to different Fisher information [22,24], but for the purposes of this paper, we will only consider some of them.

**Figure 6:**
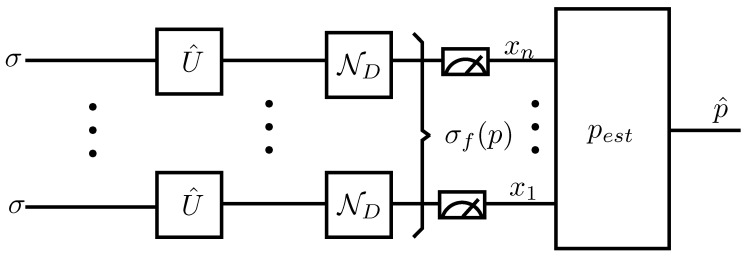
Estimation protocol used in this paper. *n* independent channel invocations are performed in order to estimate the value of *p*.

1. Number of channel invocations: For our encoding-decoding system in Figure 1, sequential channel invocations are not considered. The reason is that once a quantum state goes through the operation of the depolarization channel, it cannot be sent again through the channel. Therefore, the number of channel invocations will be set to m=1.
2. Unitary ${\hat{U}}_{1}$ transformation: The goal of the unitary transformation, ${\hat{U}}_{1}$, applied to the *n* input quantum probes, σ, is to introduce correlations among the quantum probes. In the particular case where the transformation is diagonal, it results in independent instances of the quantum probes, i.e., in an independent channel use protocol. Figure 6 shows such an estimation protocol.
  Furthermore, if *n* is set to one, the above scheme reduces to the simplest estimation protocol, called single-qubit, single-channel (SQSC). If we denote by $J_{1}\left(p\right)$ the Fisher information of this SQSC estimation protocol, then for any *n* greater than one, it can be shown [22] that the corresponding overall Fisher information $J_{n}\left(p\right)$ is given by $J_{n}\left(p\right)=nJ_{1}\left(p\right)$. Therefore, for *n* channel invocations, the quantum Cramér–Rao bound is:
  (7) $$\mathrm{var}\left(\hat{p}\right)\geq \frac{1}{nJ_{1}\left(p\right)},$$
  so that the variance bound decreases linearly with *n*. In this section, we will use the estimation protocol shown in Figure 6.
3. Input probe σ: Two different state probes σ are considered (We consider noiseless probes that are only affected by the depolarizing channel. Research about constructing robust quantum probe states to face such adverse noise has been addressed in [25,26].).
  - Unentangled pure states: The Fisher information for unentangled pure states as probes has been calculated in [27] to be:
    (8) $$J_{1}\left(p\right)=\frac{9}{8p(3-2p)}\;\;\mathrm{and}\;\;J_{n}\left(p\right)=n\frac{9}{8p(3-2p)}.$$
    It should be mentioned that for SQSC protocols with no entanglement available, (8) is the largest value that the Fisher information can attain. In other words, under pure quantum state probes, the scheme in Figure 6 is optimal.
  - Maximally entangled pure states: When entanglement is available, maximally entangled pure states or EPR pairs $|\Phi^{+}\rangle =(|00\rangle +|11\rangle )/\sqrt{2}$ can be used as probes for the depolarizing channel. It can be shown that if just one of the qubits of $|\Phi^{+}\rangle =(|00\rangle +|11\rangle )/\sqrt{2}$ is transformed by the depolarizing channel (i.e., the EPR pair goes through an extended channel $N_{D}⊗\mathrm{id}$), then the corresponding Fisher information (Note that the expressions in [24,27] are given for the depolarizing channel defined as (1). Here, we use the relationship $p=\frac{3}{4}\epsilon$ to adapt such expressions for the depolarizing channel defined as (2).) is [24]:
    (9) $$J_{1}\left(p\right)=\frac{9}{16p(1-p)}\;\;\mathrm{and}\;\;J_{n}\left(p\right)=n\frac{9}{16p(1-p)}.$$
    Note that this type of protocol requires that one of the entangled qubits is not affected by noise. This is not an issue in our scenario, since the codes we consider are entanglement assisted (there is pre-shared entanglement between the coder and the decoder), and thus, this protocol is suitable for the estimation of the depolarizing probability. It can be shown that the Fisher information value in (9), higher than in (8) due to entanglement, is the largest value that can be achieved by SQSC estimation protocols for the depolarizing channel [22].
  Although the Fisher information given by the maximally entangled probes (9) is larger than the one for pure states (8), entanglement consumption is an expensive resource, and thus, a tradeoff between performance and implementation complexity exists for this kind of estimation protocol.

#### 4.1.2. Computation of the Average Word Error Rate

Based on the results of Section 3 regarding the variation of the WER as a function of the mismatched depolarizing probability $\mathrm{WER}(\hat{p})$ (refer to Figure 3), one can obtain the average $\tilde{\mathrm{WER}}\left(p\right)$ versus the actual depolarization probability *p* of the channel as:

(10) $$\tilde{\mathrm{WER}}\left(p\right)=\int \mathrm{WER}\left(\hat{p}\right)P\left(\hat{p}\right)d\hat{p},$$

where $P(\hat{p})$ is the probability density function of the estimator being considered (Note that both $\mathrm{WER}(\hat{p})$ and $P(\hat{p})$ are functions of the true depolarizing probability *p*.). As in [12], this probability density will be assumed to be a truncated normal distribution between zero and one with the mean equal to the true depolarizing probability *p* and variance ${\left[J_{n}\left(p\right)\right]}^{-1}$, that is (We define $\mathrm{TN}(a,b,\mu ,\sigma^{2})$ as the truncated normal distribution between *a* and *b* with mean μ and variance $\sigma^{2}$.) $\hat{p}∼\mathrm{TN}\left(0,1,p,{\left[J_{n}\left(p\right)\right]}^{-1}\right)$. The selection of the inverse of the Fisher information as the variance of the distribution of the estimator $P(\hat{p})$ will give us a bound on the best performance that the system will asymptotically attain. We followed the reasoning of [22], that is as the Fisher information is asymptotically achievable, this will quantify the best possible accuracy of the quantum estimation process, giving us a bound on the performance.

**Figure 7:**
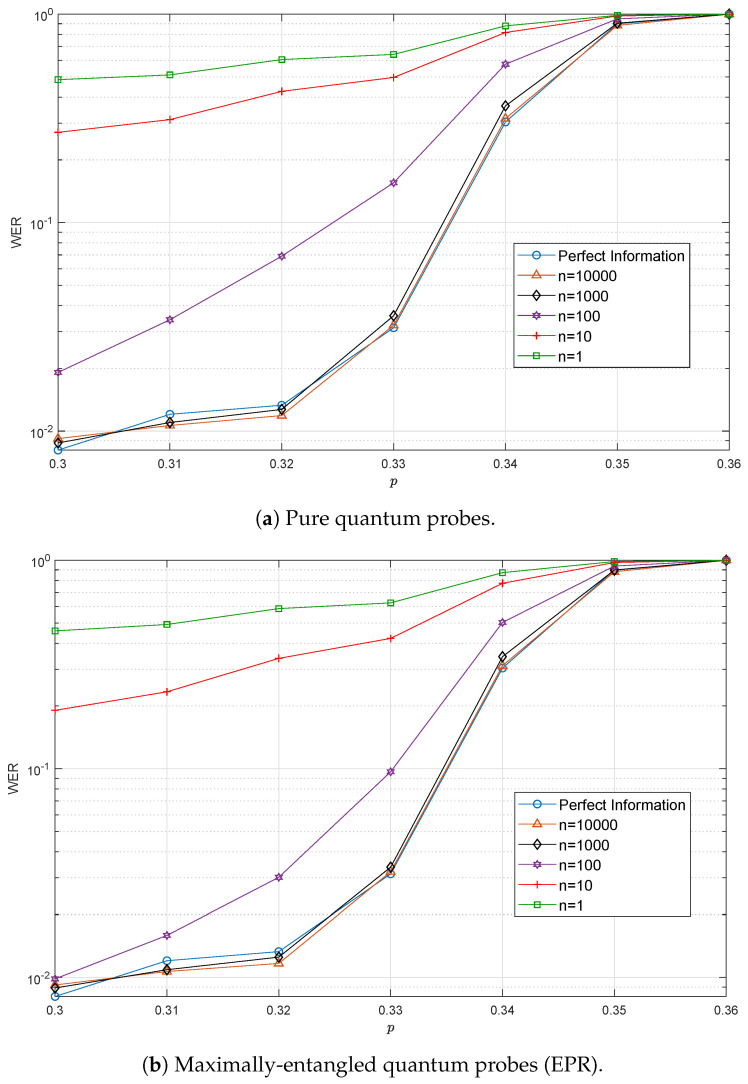
WER degradation for the QTCs as a function of the number of probes, *n*, used for the initial depolarizing probability estimation. Note that the number of probes used applies in the asymptotic setting. (**a**) Estimation using pure quantum probes. (**b**) Estimation using maximally-entangled quantum probes (EPR pairs).

Figure 7 shows the performance results of the QTCs considered in this paper versus *p* as a function of the number of probes, *n*, used. In particular, Figure 7a,b shows the results for pure state probes and for maximally entangled state probes, respectively. In both cases, the estimation protocol converged after n≈1000. However, for n=100, the protocol based on EPR pairs converged faster than the pure protocol. This agrees with the fact that the variance of the entanglement probe estimator was smaller than the variance of the pure probe estimator (refer to Expressions (8) and (9)). The drawback is that pre-shared entanglement was needed, which is an expensive resource.

### 4.2. On-Line Estimation Framework

In this section, we propose a modified version of the turbo decoding algorithm presented in Section 2.2 (described in detail in [1]) that will allow estimating the depolarizing probability jointly with the decoding process. Contrary to what happened in the off-line scheme considered in Section 4.1, this on-line estimator has the advantage of not requiring channel estimation before transmission begins. Thus, latency and rate reduction are avoided even when the quantum channel is block-to-block time varying.

As mentioned in Section 2.2 (described in detail in [1]), at each iteration *j* of the turbo decoder, the inner decoder computes the probability distribution (Note that $P_{2}^{e}\left(L_{2}\right)$ also depends on iteration *j*, but such dependency is usually omitted for simplicity.) $P_{2}^{e}\left(L_{2}\right)$ of the logical errors $L_{2}$ associated with the inner convolutional code and the probability distribution $P^{\left(j\right)}\left(P_{2}^{i}|R_{2}\right)$ that the *i*th qubit of the transmitted block will endure a physical error conditioned on the measured syndrome. Note that although $P_{2}^{e}\left(L_{2}\right)$ is sent to the outer SISO decoder through the interleaver, $P^{\left(j\right)}\left(P_{2}^{i}|R_{2}\right)$ is left unused. Such information may effectively be used to estimate the value of the depolarizing probability of the channel at decoding iteration *j* as:

(11) $${\hat{p}}^{\left(j\right)}=1-\frac{1}{N}\sum_{i=1}^{N}P^{\left(j\right)}\left(P_{2}^{i}=I|R_{2}\right),$$

where *N* is the blocklength transmitted through the channel and *I* is the identity operator.

**Figure 8:**
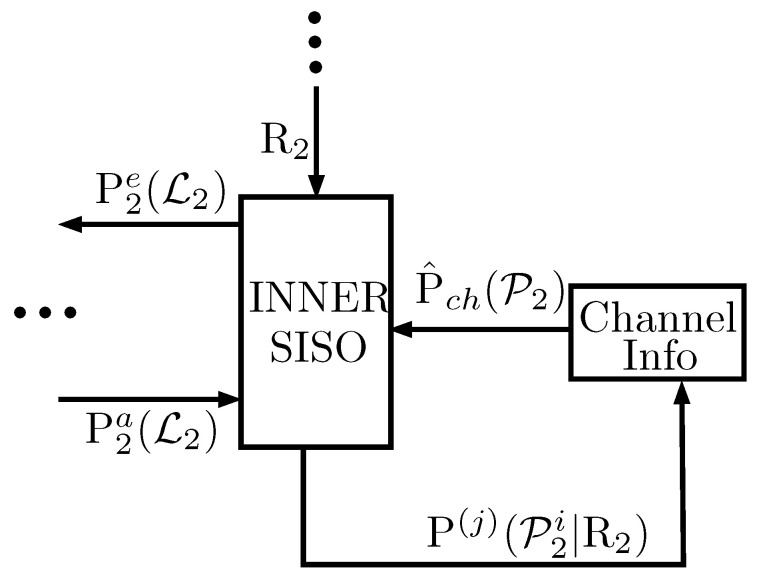
Modified QTC decoder to perform on-line estimation of the depolarizing probability. The figure only presents the inner SISO part of the decoder, as the rest of the turbo decoder remains unchanged.

Once ${\hat{p}}^{\left(j\right)}$ is obtained, it is fed to the inner SISO decoder. Under the low WER decoding operation, it is expected that the sequence of estimates $\left\{{\hat{p}}^{\left(j\right)}\right\}$ will converge to the actual depolarization probability of the channel *p*. This procedure is schematically represented in Figure 8.

**Figure 9:**
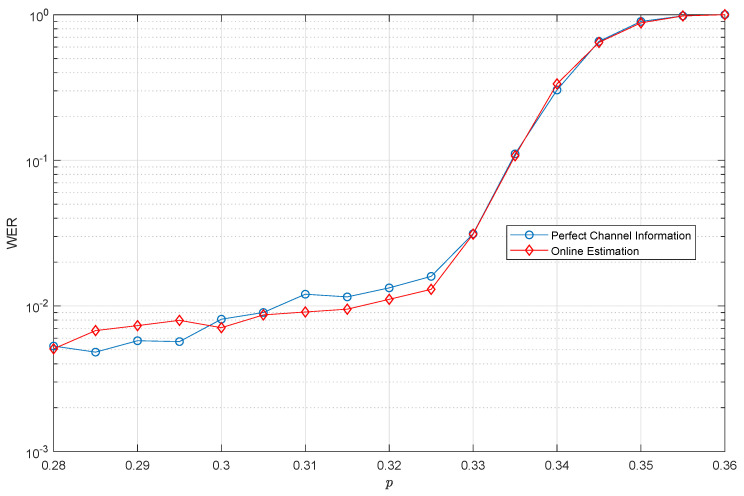
WER performance for the QTCs when the proposed on-line estimation procedure for the depolarizing probability is utilized with initial value ${\hat{p}}^{\left(1\right)}=p^{*}=0.3779$. For comparison purposes, the figure also shows the WER achieved when perfect channel information is available.

A question that remains to be answered is what criterion one should follow to provide the initial value of the depolarization probability, ${\hat{p}}^{\left(1\right)}$, to the inner SISO decoder. The selection of the initial value may affect the convergence rate to the final value of *p*, and in turn the decoder operation. Figure 9 presents the results when the initial value, ${\hat{p}}^{\left(1\right)}$, is equal to the hashing limit of the QTC, i.e., ${\hat{p}}^{\left(1\right)}=p^{*}$. The rationale behind this selection is that no QECC with a hashing limit $p^{*}$ will be able to operate at low WER whenever the depolarization probability *p* is larger than $p^{*}$, and thus, initialization with ${\hat{p}}^{\left(1\right)}>p^{*}$ makes no sense in principle. In our case, $p^{*}=0.3779$ (refer to Figure 3).

Figure 9 shows the QTC performance in terms of WER versus *p* when using the proposed on-line estimation method with initial value ${\hat{p}}^{\left(1\right)}=p^{*}=0.3779$. For comparison purposes, the figure also shows the WER achieved when perfect channel information is available to the inner SISO decoder. Notice that there was no WER degradation when using on-line estimation, since the turbo code with the on-line estimation of the depolarizing probability achieved the same error correction capability as when the channel information was perfectly available at the decoder. Importantly, this performance was achieved without requiring extra qubits and, therefore, without decreasing the rate of the QTC, since the information used for this on-line protocol was based on information that the SISO decoding algorithm already calculated.

**Figure 10:**
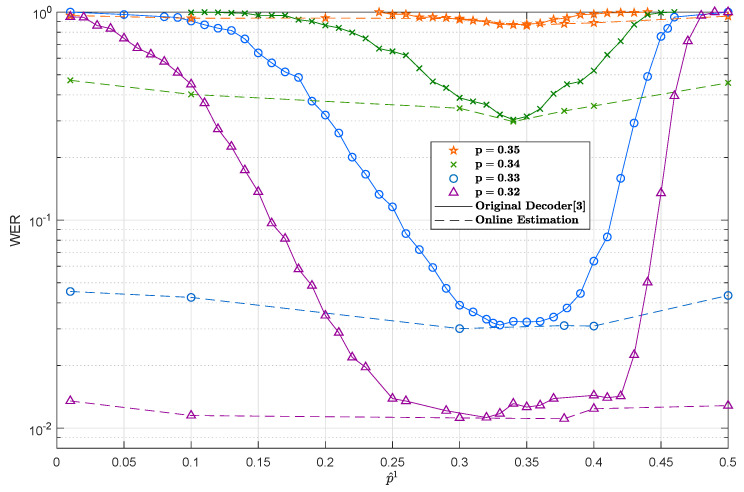
WER variation for the QTCs as a function of the initial value of the depolarizing probability, ${\hat{p}}^{\left(1\right)}$. The continuous lines represent the sensitivity of the original turbo decoder [1] (no estimation of the depolarizing probability is performed, and the decoder always utilizes ${\hat{p}}^{\left(1\right)}$), while the dashed lines represent the sensitivity of the modified decoder using the proposed on-line estimation method.

In order to assess the effect of the choice of the initial value of the depolarizing probability, Figure 10 depicts the WER performance when ${\hat{p}}^{\left(1\right)}$ is chosen as $\hat{p}$ in the *x*-axis. Each curve corresponds to a different actual value of the depolarizing probability, *p*. For comparison purposes, the curves in Figure 3 are also depicted here (in that case, $\hat{p}$ is not estimated jointly with the decoding process). Notice the excellent performance achieved by the on-line estimation method independently of the initial value ${\hat{p}}^{\left(1\right)}$, which manifests by the much flatter nature of the curves obtained by the on-line estimation method when compared with those of Figure 3.

## 5. Conclusions

We studied the performance sensitivity of QTCs applied over depolarizing channels when there exists a mismatch between the actual depolarizing probability and the probability fed to the decoders. To that end, we fed the turbo decoder with values different from the actual value of the depolarizing probability and observed the degradation in the WER due to such mismatch. Simulation results showed that the decoder was sensitive both to under- and over-estimation of the depolarizing probability, presenting a flat region around the actual value of the depolarizing probability. In order to overcome the lack of knowledge regarding the depolarization probability, we proposed a novel on-line estimation procedure based on the information generated in the iterative turbo decoder. Such an on-line estimator outperformed existing off-line estimators in terms of overall coding rate and latency, while maintaining excellent WER performance. Simulation results showed that the on-line estimation method was insensitive to the initial value of the depolarizing probability, and thus, the resulting performance experienced very little degradation in the presence of channel mismatch.

## Figures and Tables

**Table 1:** Parameters of the QCC encoders.

Config.	Encoder	R	E	m	Seed Transformation U
EXIT- optimized	Outer	1/3	0	3	{1048,3872,3485,2054, 983,3164,3145,1824, ${987,3282,2505,1984\}}_{10}$
	Inner	1/3	2/3	3	{4091,3736,2097,1336, 1601,279,3093,502, ${1792,3020,226,1100\}}_{10}$

*R* and *E* refer to the coding rate and the entanglement consumption rate, respectively. *m* refers to the memory qubits. The seed transformations U are represented using the decimal representation presented in [4].

## Abbreviations

The following abbreviations are used in this manuscript:

QECC	Quantum error correction code
QCC	Quantum convolutional code
QTC	Quantum turbo code
QLDPC	Quantum low density parity check
QIRCC	Quantum irregular convolutional code
QURC	Quantum unity rate code
EXIT	Extrinsic information transfer
EPR	Einstein–Podolsky–Rosen
SISO	Soft-input soft-output
JPL	Jet Propulsion Laboratory
WER	Word error rate
SQSC	Single-qubit single-channel
SLD	Symmetric logarithmic derivative
